# Real-World Management of Patients with Primary Biliary Cholangitis—A Retrospective Study from a Tertiary Medical Center in Israel

**DOI:** 10.3390/jcm10194551

**Published:** 2021-09-30

**Authors:** Eyal Yehezkel, Inbal Israel, Inbal Houri, Moshe Leshno, Oren Shibolet, Ehud Zigmond

**Affiliations:** 1 Center for Autoimmune Liver Diseases, Department of Gastroenterology, Tel Aviv Sourasky Medical Center, Tel Aviv 6423906, Israel; eyalyehez@gmail.com (E.Y.); inbalho@tlvmc.gov.il (I.H.); leshnom@tauex.tau.ac.il (M.L.); orensh@tlvmc.gov.il (O.S.); 2 Sackler Faculty of Medicine, Tel Aviv University, Tel Aviv 6997801, Israel; inbal264@gmail.com

**Keywords:** primary biliary cholangitis, ursodeoxycholic acid, treatment response, prognosis

## Abstract

Background: Primary biliary cholangitis (PBC) is a rare autoimmune liver disease with variation in prevalence, phenotype and prognosis across different geographical regions. Little is known about PBC in Israel. Our aim was to characterize the demography, clinical presentation, treatment patterns and prognosis in a cohort of PBC patients followed in a referral center in central Israel. Methods: Clinical, demographic and laboratory data were collected from the medical records of PBC patients followed at Tel Aviv Medical Center in the years 2003–2020. Results: We have identified 189 patients with a confirmed diagnosis of PBC; 92.6% were female and the mean age at diagnosis was 54.7 years. Thirty-nine percent were diagnosed with another autoimmune disease and 5.9% were diagnosed with a PBC-AIH (autoimmune hepatitis) variant syndrome. Ninety-six percent were treated with ursodeoxycholic acid (UDCA) at a mean dose of 13.3 mg/kg. A total of 28.1% were found with inadequate response to UDCA according to the Toronto criteria, and 53% of the UDCA non-responders were treated with bezafibrate. Younger age at diagnosis, higher baseline levels of alkaline phosphatase (ALP) and gamma-glutamyltransferase (GGT), AIH-PBC variant and positive anti-smooth muscle antibodies (ASMA) were associated with an inadequate UDCA response. In a multivariable analysis, higher ALP at diagnosis (OR = 1.92 CI 1.11–3.20 per 50-unit change, *p* = 0.018) and ASMA (OR = 27.6 CI 2.58–295, *p* = 0.006) independently predicted inadequate UDCA response. Higher alanine transaminase (ALT), ALP and GGT, lower albumin, younger age at diagnosis and pruritus conferred an increased risk for disease progression. Conclusions: Disease characteristics, treatment patterns, response to therapy and prognosis of a PBC patient cohort in a tertiary center in central Israel were revealed. The results highlight the importance of risk stratification in PBC, specifically in younger patients, those presenting with a high level of liver enzymes and in ASMA-positive patients with an assumed diagnosis of the AIH-PBC variant.

## 1. Introduction

Primary biliary cholangitis (PBC) is a chronic autoimmune liver disease of the small intrahepatic bile ducts. The disease typically affects middle aged women, with a female to male ratio of up to 10:1 [1], although a higher prevalence in males and younger age at diagnosis has been recently reported [2,3]. PBC is characterized by a cholestatic pattern of serum liver enzymes, the presence of anti-mitochondrial antibodies (AMA) and/or the PBC specific anti-nuclear antibodies (ANA) (anti-sp100 and anti-gp210), and typical histological features [4]. Without treatment, PBC may progress to cirrhosis and hepatic failure, and eventually, may require liver transplantation.

Ursodeoxycholic acid (UDCA) therapy was shown to improve liver-transplant-free survival and is considered the cornerstone of treatment [5,6]. Until recently, UDCA was the only therapeutic option for PBC patients; however, several new treatment options have recently been added to the treatment armamentarium as adjuncts to UDCA, in patients with inadequate response. The availability of these second line drugs, obeticholic acid and fibrates, varies greatly between different geographical regions.

Several biochemical UDCA response criteria have been developed in order to predict prognosis, either binary (Paris I, Paris II, Rotterdam, Toronto or Barcelona) or continuous (GLOBE score [7] and the UK-PBC risk score [8]). The biochemical response rates to UDCA vary widely according to the exact criteria and the population [9,10]. Notably, liver-transplantation-free survival is markedly decreased in patients with poor UDCA response [8] and current guidelines emphasize the importance of timely assessment of patients at high risk without adequate response to first line treatment [11].

PBC is considered a rare liver disease; however, its incidence rate is increasing worldwide [12]. In 2018, its prevalence in the US was reported to be 29.3 per 100,000. In a recent meta-analysis, the prevalence and annual incidence in Europe from 2000 through to 2020 were 22.3 and 1.87 per 100,000, respectively [13]. In Israel, the epidemiological data are limited. In 2012, Delgado et al. reported a prevalence of 25.5 cases per 100,000 in southern Israel [14]. Population-based studies showed a wide range of incidence and prevalence of PBC throughout the world [15]. Recently, a latitudinal geoepidemiological pattern of occurrence has been proposed, with a higher prevalence in northern countries [16]. Interestingly, the rates of non-response to UDCA also varied in different studies depending on the geographical region and the study population [17]. For example, the inadequate response rate according to the Toronto criteria were found to be as high as 38.6% in a single center in England and 33% in a population study in Iceland [18,19], while in a single center in China and in a multicenter study in Italy, lower non-responder rates of 17% and 20.1%, were reported, respectively [17,20].

The aim of the present study was to characterize the PBC population in a large tertiary referral medical center in Tel Aviv, Israel. We aimed to characterize disease phenotype including demographic data, disease-related symptoms and severity, laboratory parameters, presence of concomitant medical disorders, treatment patterns, treatment response rates and prognosis. Additionally, we evaluated associations of biochemical and demographical variables and UDCA response, and investigated prognostic factors for clinical endpoint of advanced liver disease and its complications.

## 2. Materials and Methods

This retrospective, single-center study included patients who were diagnosed with PBC according to ICD-9 coding (The International Classification of Diseases, ICD-9 code 571.6) and began follow-up in the outpatient liver clinic at Tel Aviv Sourasky Medical Center, during the period of January 2003–May 2020. Clinical, demographic and laboratory data were collected from electronic medical records. PBC diagnosis was defined according to European Association for the Study of the Liver (EASL) guidelines [11]. Two senior hepatologists with specific expertise in autoimmune and cholestatic liver diseases evaluated the data independently and decided which patients were correctly diagnosed with PBC and the PBC-AIH (autoimmune hepatitis) overlap variant, and who to exclude (non-agreement was discussed and agreed by consensus). Patients not meeting the diagnostic criteria or with an alternative diagnosis were excluded. Clinical endpoint was defined as any of the following clinical events: diagnosis of liver cirrhosis, liver transplantation, hepatocellular carcinoma, liver-related death, variceal bleeding, spontaneous bacterial peritonitis, hepatic encephalopathy, ascites, and hepatorenal syndrome. The degree of fatigue and pruritus was assessed by the treating physician during the outpatient clinic visit, and classified as severe or mild to moderate. Histological reports of liver biopsies were reviewed and assessed for the presence of interface hepatitis and the fibrosis stage. Advanced fibrosis was defined as Ludwig’s stages 3 or 4 (bridging fibrosis or cirrhosis).

The study was approved by the Sourasky Medical Center’s institutional ethics committee in keeping with the principles of the Declaration of Helsinki, approval number 0163-20. An informed consent waiver was granted by the IRB committee as the data were recorded anonymously.

Statistical Analysis: Data are presented as means and standard deviations for continuous variables. For analysis of UDCA response, we estimated the odds ratios (OR) by conducting a univariable logistic regression and, in addition, we conducted a stepwise logistic regression for multivariable analysis with entry probability of 0.1 and removal probability of 0.15. For analysis of clinical endpoint, we estimated the hazard ratios (HR) with the use of Cox regression models. In addition, comparing the UDCA responders and the UDCA non-responders groups, we applied the log-rank test and the Cox model and presented it in a Kaplan–Meier curve. *p*-value < 0.05 was considered statistically significant. We used MATLAB software, version R2020b (MathWorks, Natick, MA, USA), and IBM SPSS version 27 (IBM, Armonk, NY, USA) for all the statistical analyses.

## 3. Results

### 3.1. Patients and Disease Characteristics

**Figure 1:**
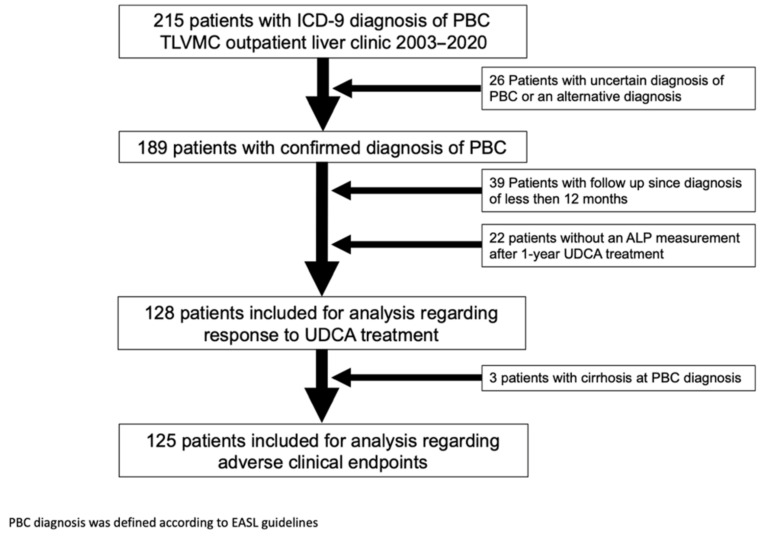
Study flow diagram. ICD, The International Classification of Diseases; PBC, primary biliary cholangitis; TLVMC, Tel Aviv Sourasky Medical Center; UDCA, ursodeoxycholic acid; ALP, alkaline phosphatase; EASL, European Association for the Study of the Liver.

A total of 215 patients with a diagnosis of PBC according to ICD-9 coding were found in a digital database search for patients who visited the outpatient liver clinic of the gastroenterology department in Tel Aviv Medical Center between January 2003 and May 2020. Revision of patients’ medical records found 26 patients without a probable diagnosis of PBC according to EASL guidelines [11] or with an alternative diagnosis; thus, a total of 189 patients with PBC were included for further analysis (Figure 1).

Among patients, 175 (92.6% of the cohort) were females and the average age at diagnosis was 54.7 years (±13.0). In total, 86.5% of the patients (147/170) were AMA-positive, 75.5% had at least one positive test for anti-nuclear antibodies (ANA) and 13.5% for anti-smooth muscle antibodies (ASMA). In total, 72.3% of the patients (125/173) were Ashkenazi Jews, and 24.9% (43/173) were Sephardi Jews; 39.2% of the patients (73/186) were diagnosed with at least one other autoimmune disease, most commonly hypothyroidism (19.9% of patients). The AIH-PBC overlap variant syndrome was diagnosed in 11 patients (5.9%). Twenty-eight patients (15.1%) had a history of cholecystitis or cholecystectomy (Table 1).

Most of the patients (96.3%, 182/189) were treated with UDCA with a mean dose of 13.3 (±4.47) mg UDCA/kg BW/d. Regarding the use of additional medications for PBC, 18.5% of the patients (35/189) were treated with bezafibrate, 13.8% (26/189) were treated with corticosteroids (prednisone or budesonide), and 2.65% (5/189) with obeticholic acid. Assessment of disease progression in the entire cohort revealed that 17.5% (33/189) of the patients reached an adverse clinical endpoint. Of those, 15.9% (30/189) developed cirrhosis, 5.3% (10/189) ascites, 3.7% (7/189) hepatic encephalopathy, 3.2% (6/189) variceal bleeding, 1.6% (3/189) hepato-renal syndrome, 0.5% (1/189) spontaneous bacterial peritonitis, and 0.5% (1/189) liver-related death. 7 patients, comprising 3.7% of the cohort, underwent liver transplantation.

### 3.2. Response to UDCA Treatment

Patients with a follow-up shorter than 12 months were excluded (Figure 1). Biochemical response to UDCA was evaluated according to accepted criteria (Table 2).

UDCA non-response rates were found in the range of 16.7–40.6% of the patients according to different dichotomous criteria (Table 2). As the Toronto criteria are based solely on ALP level, we had more patients with available information for this criterion and, thus, we chose this criterion for further statistical evaluations. Within the 128 patients included in the analysis, the median follow-up time since PBC diagnosis was 7 years with an interquartile range of 10 years. Univariable analysis for association of baseline disease parameters with UDCA non-response (Toronto criteria) revealed that patients with younger age and higher baseline ALP and GGT at diagnosis were less likely to respond to UDCA therapy with an OR = 0.95 CI 0.92–0.99 per year, *p* = 0.009, OR = 1.38 CI 1.15–1.66 for a 50-unit change, *p* < 0.001 and OR = 1.02 CI 1–1.04 for a 10-unit change, *p* = 0.03, respectively (Table 3).

The PBC-AIH variant syndrome was diagnosed in 11 patients (5.9% of the cohort) and ASMA was found positive in 17 patients (13.5% of the cohort; Table 1); both were associated with reduced response rates to UDCA therapy with an OR of 4.78 CI 1.08–21.2, *p* = 0.039, and OR = 8 CI 1.92–33.26, *p* = 0.004, respectively, (Table 3). Liver histology results were available for 52 patients. The presence of interface hepatitis was associated with reduced response rates to UDCA therapy with an OR of 12 CI 1.43–100.8, *p* = 0.022, while advanced fibrosis was not statistically significantly associated with response rates to UDCA (Table 3). No significant association with UDCA response rates was found for gender, ethnicity, symptoms of fatigue or pruritus, presence of other autoimmune diseases and serology of AMA or ANA. In a multivariable analysis, higher ALP at diagnosis (OR = 1.92 CI 1.11–3.20 per 50-unit change, *p* = 0.018) and ASMA (OR = 27.6 CI 2.58–295, *p* = 0.006) independently predicted inadequate UDCA response.

### 3.3. Risk Factors Associated with Disease Progression

**Figure 2:**
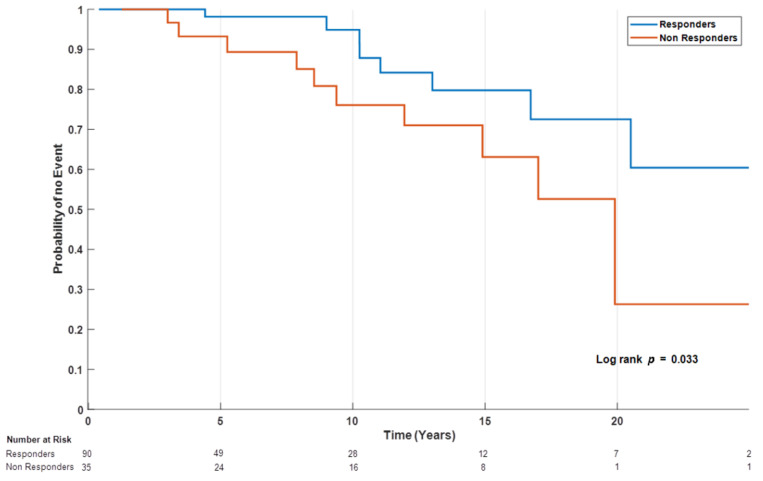
Kaplan–Meier survival curve to achieve any clinical endpoint in UDCA responder (blue line) versus non-responders (red line).

One hundred and twenty-eight patients had a follow up period of at least 12 months and information regarding response to UDCA therapy (Figure 1); of these, 23 reached a clinical endpoint. Overall, 21 patients developed liver cirrhosis, 9 of them with decompensation. Of note, three patients were already diagnosed with liver cirrhosis at PBC diagnosis. As expected, UDCA non-responders were more likely to reach a clinical endpoint with 31.4% versus 10%, in UDCA non-responder versus responders, respectively (*p* = 0.033 log rank test) (Figure 2).

Univariable Cox regression analysis revealed an increased risk for reaching a clinical endpoint in patients with baseline higher ALT (HR 1.17 CI 1.02–1.35 per 10-unit change, *p* = 0.026), ALP (HR 1.28 CI 1.04–1.58 per 50-unit change, *p* = 0.022) and GGT (HR 1.03 CI 1.00–1.06 per 10-unit change, *p* = 0.021), as well as in patients with lower serum albumin (HR 0.93 CI 0.86–1.00, *p* = 0.044) and in patients with younger age at diagnosis (HR 0.96 CI 0.93–1.00, *p* = 0.046) (Table 4).

Interestingly, patients who suffered from pruritus had an increased risk of reaching a clinical endpoint (HR 3.45 CI 1.29–9.22, *p* = 0.014). Gender, fatigue, and the presence of other liver diseases or other autoimmune diseases were not significantly associated with early clinical endpoint. Due to lack of statistical power, we could not calculate odds ratios for histological parameters. Multivariable analysis did not identify statistically significant parameters associated with adverse clinical outcome.

## 4. Discussion

PBC is an autoimmune liver disease with a reported geographical variability in prevalence, disease phenotype and response to treatment. Moreover, second line treatments beyond UDCA have emerged in recent years but still greatly vary between countries depending on local health regulations, drug availability and insurance coverage. Little is known about PBC in Israel and here, we present data on two decades of follow up of PBC patients in a tertiary liver center in central Israel.

Patients’ characteristics and baseline lab values in our study (Table 1) represent a typical PBC cohort with predominant female gender, cholestatic pattern of liver enzymes and a normal mean value of total bilirubin, albumin and platelets. Interestingly, we have found that about three-quarters of the cohort had positive ANA, while a recent study that included 434 PBC patients showed a lower ANA prevalence of about 55% [21]. The higher prevalence of ANA in our study may characterize the specific Jewish Israeli population in our study. Alternatively, this difference could be related to the fact that many of the patients in our cohort had blood tests performed in several different labs in primary care settings and in hospitals that use different techniques for ANA detection.

Biochemical non-response to UDCA is a well-known predictor of long-term histological progression [10]. UDCA response rates in our cohort were found to be generally similar to previous reports, yet higher in comparison to recent similar studies [21,22]. According to the Barcelona criterion, we have documented a non-response rate of 40.6%, while multicenter data from Portugal and Germany reported rates of 30.7% and 38.7%, respectively [21,22]. In addition, when assessing response rates according to the Toronto criterion, we found a non-response rate of 28.1%, while a recently published German study found a non-response rate of only 16.2% according to this criterion [22]. These higher percentages of UDCA non-response in our study could be attributed to the accumulation of difficult to treat patients in a referral tertiary center [17].

Eleven patients, representing 5.9% of the cohort, were diagnosed with the AIH-PBC variant syndrome. Interestingly, we found that the presence of PBC-AIH variant syndrome as well as the presence of ASMA conferred an increased risk of UDCA non-response with HRs of 4.8 and 8, respectively. Moreover, positive ASMA predicted lower response rates to UDCA independently in a multivariable analysis. These findings are in agreement with a recent study demonstrating reduced response rates to UDCA in PBC patients with autoimmune hepatitis features [23]. Of note, a previous report showed that the PBC-AIH variant was associated with higher rates of cirrhosis complications when compared to PBC or AIH alone [24]. Our findings suggest that these patients are at increased risk for complications and may benefit from additive therapeutic strategies.

We found that young age at diagnosis was a major risk factor for UDCA non-response. This association, which probably represents a more aggressive disease course in the younger population, has been described recently in large PBC cohorts [9,25,26]. In addition, in partial agreement with previous reports, we found ALP and GGT levels at baseline to predict inadequate UDCA response [21,26,27,28]. Liver biopsy is not mandatory for the diagnosis or follow up in PBC [11]; nevertheless, histological data were available in this study for 52 out of the 125 patients included in the analysis. Interface hepatitis, but not advanced fibrosis, was found to be associated with UDCA non-response. In fact, the absence of interface hepatitis was found to be a strong predictor of biochemical response to UDCA as only 1 out of 16 patients without interface hepatitis failed to respond to UDCA treatment, thus corroborating a previous study that addressed this issue [29].

Regarding risk factors for disease prognosis, we found younger age at diagnosis, high levels of ALT, GGT and ALP and low level of albumin at baseline to be associated with disease progression; all were shown in previous studies to predict a worse prognosis in PBC patients [7,8,30]. In addition, we found pruritus to be associated with reaching of a clinical endpoint. Notably, a few patients in our cohort underwent liver transplantation due to severe pruritus, without end-stage liver disease.

This study has several limitations. First, it is retrospective and prone to limitations associated with this design. Second, we reviewed the medical records of patients within the PBC clinic at a single referral hospital in Tel Aviv; therefore, our cohort may not represent PBC patients in the general Israeli population. Third, the number of patients with documented adverse clinical events was relatively small, thus hampering the statistical power of the study, especially the multivariable analysis.

In conclusion, we describe a large cohort of PBC patients in a referral liver center in central Israel. An extensive analysis of disease characteristics, management patterns, response to therapy and prognosis has been explored. The data revealed baseline parameters associated with inadequate response to therapy and prognosis, further strengthening the importance of risk stratification in the management of patients with PBC.

## Figures and Tables

**Table 1:** Patients, disease characteristics, laboratory values, treatment patterns and clinical endpoints.

Number of Patients		189
Demographics	Female gender, *n* (%)	175 (92.6)
	Age at diagnosis, years, mean (std)	54.7 (13.0)
	Ethnicity, *n* (%)	
	Jewish Ashkenazi	125 (72.3)
	Jewish non-Ashkenazi	43 (24.9)
	Other	5 (2.9)
	Smoking, *n* (%)	31 (23.1)
Symptoms	Fatigue, *n* (%) Severe fatigue, *n* (%)	80 (47.9) 28 (14.8)
	Pruritus, *n* (%) Severe pruritus, *n* (%)	56 (29.6) 21 (11.1)
Other liver diseases	NASH, *n* (%)	37 (20.0)
	Cholecystitis/Cholecystectomy, *n* (%)	28 (15.1)
	PBC-AIH variant, *n* (%)	11 (5.9)
	Chronic HBV, *n* (%)	2 (1.1)
Other autoimmune diseases	Any extrahepatic autoimmune disease, *n* (%)	73 (39.2)
	Hypothyroidism, *n* (%)	37 (19.9)
	Celiac, *n* (%)	3 (1.6)
	Scleroderma, *n* (%)	4 (2.2)
	Sjogren, *n* (%)	11 (5.9)
	Rheumatoid arthritis, *n* (%)	9 (4.8)
	Psoriasis, *n* (%)	4 (2.2)
	Systemic lupus erythematosus, *n* (%)	2 (1.1)
	Ulcerative colitis, *n* (%)	3 (1.6)
	Immune thrombocytopenic purpura, *n* (%)	4 (2.2)
	Raynaud’s syndrome, *n* (%)	3 (1.6)
	Other autoimmune disease, *n* (%)	8 (4.3)
Baseline Laboratory results (before UDCA) mean (std)	Hemoglobin (g/L)	13.0 (1.2)
	Platelets (×10^9^/L)	237 (80)
	Creatinine (mg/dL)	0.78 (0.22)
	Total bilirubin (mg/dL)	0.74 (0.56)
	AST (IU/L)	59.2 (43.1)
	ALT (IU/L)	72.7 (66.8)
	ALP (IU/L)	280 (200)
	GGT (IU/L)	342 (339)
	Albumin (g/L)	40.3 (5.5)
	IgM (mg/dL)	418 (382)
Immune Serology	AMA, *n* (%)	147 (86.5)
	ANA, *n* (%)	108 (75.5)
	ASMA, *n* (%)	17 (13.5)
	Anti-gp210, *n* (%)	9 (32.1)
	Anti-sp100, *n* (%)	8 (29.6)

Std, standard deviation; NASH, non-alcoholic steatohepatitis; PBC-AIH, primary biliary cholangitis-autoimmune hepatitis; HBV, hepatitis B virus; AST, aspartate transaminase; ALT, alanine transaminase; ALP, alkaline phosphatase; GGT, gamma-glutamyltransferase; IgM, immunoglobulin M; AMA, anti-mitochondrial antibodies; ANA, anti-nuclear antibodies; ASMA, anti-smooth muscle antibodies.

**Table 2:** UDCA non-response rates according to different criteria.

UDCA Biochemical Response Rates			
Criteria	Definition of Biochemical Response	*n*	Non-responder Rate
Toronto	ALP ≤ 1.67 × ULN	128	28.1%
Barcelona	ALP normalization or decrease >40%	69	40.6%
Paris-I	ALP ≤ 3 × ULN and AST ≤ 2 × ULN and normal total bilirubin	86	17.4%
Paris-II	ALP ≤ 1.5 × ULN and AST ≤ 1.5 × ULN and normal total bilirubin	86	38.4%
Rotterdam	Albumin and total bilirubin normalization	96	16.7%
GLOBE score	Below age-specific threshold	81	17.3%
UK PBC score	Risk of event in 5 years < 5%	79	6.3%

UDCA, ursodeoxycholic acid; ULN, upper limit of normal.

**Table 3:** Univariable analysis for parameters associated with UDCA non-response using Toronto criteria.

		Responder	Non-Responder	Univariable Analysis	
	Number of Patients, *n* (%)	92 (71.9)	36 (28.1)	OR (95% CI)	*p-*Value
Demographics	Female gender, *n* (%)	87 (94.6)	32 (88.9)	0.46 (0.12–1.82)	0.268
	Age at diagnosis, years, mean (std)	55.6 (12.6)	48.8 (12.6)	0.95 (0.92–0.99)	0.009
	Ethnicity, *n* (%)				
	Jewish Ashkenazi	61 (71.8)	26 (78.8)	1.3 (0.5–3.38)	0.588
	Jewish non-Ashkenazi	21 (24.7)	7 (21.2)		
	Other	3 (3.5)	0 (0)		
	Smoking, *n* (%)	13 (19.1)	7 (28.0)	1.49 (0.68–3.23)	0.316
Symptoms	Fatigue, *n* (%)	38 (43.7)	22 (62.9)	0.50 (0.22–1.10)	0.083
	Severe fatigue, *n* (%)	13 (14.1)	8 (22.2)	1.37 (0.55–3.42)	0.499
	Pruritus, *n* (%)	29 (32.6)	15 (42.9)	0.67 (0.30–1.46)	0.311
	Severe pruritus, *n* (%)	9 (9.8)	8 (22.2)	2.22 (0.84–5.85)	0.106
Other liver diseases	NASH, *n* (%)	15 (16.7)	10 (27.8)	1.97 (0.79–4.93)	0.145
	Cholecystitis/Cholecystectomy, *n* (%)	9 (10.0)	8 (22.2)	2.63 (0.93–7.49)	0.069
	PBC-AIH variant, *n* (%)	3 (3.3)	5 (13.9)	4.78 (1.08–21.2)	**0.039**
	Chronic HBV, *n* (%)	0 (0)	1 (2.8)		
Other AI diseases	Extrahepatic autoimmune disease, *n* (%)	41 (45.1)	13 (36.1)	0.7 (0.32–1.56)	0.385
	Hypothyroidism, *n* (%)	17 (18.7)	4 (11.1)	0.42 (0.13–1.33)	0.141
Baseline lab results (before UDCA) mean (std)	Hemoglobin (g/L)	12.9 (1.2)	13.2 (0.9)	1.19 (0.68–2.07)	0.545
	Platelets (× 10^9^/L)	236 (74)	234 (98)	1 (0.99–1.01)	0.945
	Creatinine (mg/dL)	0.77 (0.23)	0.76 (0.16)	0.97 (0.03–31.15)	0.984
	Total bilirubin (mg/dL)	0.62 (0.26)	1.16 (1.02)	5.84 (0.97–35.32)	0.055
	AST (IU/L) *	50.4 (32.9)	76.6 (63.6)	1.13 (0.98–1.31)	0.087
	ALT (IU/L) *	65.0 (69.4)	101.1 (85.2)	1.06 (0.98–1.14)	0.142
	ALP (IU/L) **	222 (125)	518 (309)	1.38 (1.15–1.66)	**<0.001**
	GGT (IU/L) *	275 (284)	545 (479)	1.02 (1–1.04)	**0.030**
	Albumin (g/L)	41.2 (3.2)	38.8 (10.8)	1.02 (0.82–1.27)	0.871
Immune Serology	AMA, *n* (%)	75 (88.2)	26 (78.8)	0.5 (0.17–1.44)	0.195
	ANA, *n* (%)	54 (74.0)	25 (89.3)	2.93 (0.79–10.83)	0.107
	ASMA, *n* (%)	3 (4.9)	8 (29.6)	8 (1.92–33.26)	**0.004**
	Anti-gp210, *n* (%)	2 (18.2)	4 (44.4)		
	Anti-sp100, *n* (%)	3 (27.2)	2 (25.0)		
Liver Histology	Interface Hepatitis, *n* (%)	20 (57.1)	16 (94.1)	12 (1.43–100.8)	0.022
	Advanced Fibrosis, *n* (%)	7 (21.2)	5 (27.8)	1.43 (0.38–5.38)	0.598

* denotes OR for a 10-unit change ** denotes OR for a 50-unit change. Bold values indicate a statistical significance (*p* < 0.05).

**Table 4:** Univariable analysis for reaching a clinical endpoint.

		HR (95% CI)	*p*-Value
Demographics	Female gender	1.26 (0.17–9.55)	0.826
	Age at diagnosis	0.96 (0.93–1.00)	**0.046**
	Ethnicity—Jewish Ashkenazi	2.89 (0.66–12.64)	0.159
	Smoking	0.59 (0.13–2.63)	0.487
Symptoms	Fatigue	1.37 (0.53–3.53)	0.521
	Severe fatigue	0.90 (0.26–3.16)	0.875
	Pruritus	3.45 (1.29–9.22)	**0.014**
	Severe pruritus	4.29 (1.62–11.34)	**0.003**
Other liver diseases	NASH	1.72 (0.56–5.24)	0.342
	Cholecystitis/Cholecystectomy	0.96 (0.22–4.24)	0.957
	PBC-AIH variant	1.77 (0.50–6.23)	0.374
Other AI diseases	Extrahepatic autoimmune disease	0.89 (0.35–2.28)	0.809
	Hypothyroidism	0.86 (0.28–2.60)	0.790
Laboratory before UDCA	Hemoglobin	0.18 (0.02–1.40)	0.101
	Platelets	0.99 (0.98–1.00)	0.178
	Creatinine	16.21 (0.07–3710)	0.315
	Total bilirubin	2.30 (0.89–5.96)	0.086
	AST *	1.28 (0.94–1.74)	0.115
	ALT *	1.17 (1.02–1.35)	**0.026**
	ALP **	1.28 (1.04–1.58)	**0.022**
	GGT *	1.03 (1.00–1.06)	**0.021**
	Albumin	0.93 (0.86–1.00)	**0.044**
Immune Serology	AMA	5.40 (0.71–41.21)	0.104
	ANA	2.42 (0.31–18.7)	0.396
	ASMA	2.27 (0.60–8.61)	0.227

* denotes OR for a 10-unit change ** denotes OR for a 50-unit change. Bold values indicate a statistical significance (*p* < 0.05).

## Data Availability

The data presented in this study are available on request.

## Abbreviations

PBC	Primary Biliary Cholangitis
AIH	Autoimmune hepatitis
ALP	Alkaline phosphates
GGT	Gamma glutamyl transferase
ALT	Alanine transaminase
AST	Aspartate transaminase
AMA	Anti-mitochondrial antibodies
ANA	Anti-nuclear antibodies
ASMA	Anti-smooth muscle antibodies
UDCA	Ursodeoxycholic acid
ULN	Upper limit of normal
OR	Odds ratio
HR	Hazard ratio
EASL	European Association for the Study of the Liver
